# Comparative Effectiveness of Massage Gun and Myofascial Release Technique for Releasing Latent Trigger Points of Calf Muscle: A Randomized Clinical Trial

**DOI:** 10.1002/hsr2.72703

**Published:** 2026-06-28

**Authors:** Md. Mafrohi Sattar, Abid Hasan Khan, Kazi Md Azman Hossain, Md. Feroz Kabir, Sharmila Jahan, K. M. Amran Hossain, Ehsanur Rahman, Farzana Sharmin, Md. Saruar Hossain Bhuiyan, Azharul Islam, Md. Kabir Hossain, Md. Zahid Hossain

**Affiliations:** ^1^ Department of Physiotherapy Center for the Rehabilitation of the Paralyzed (CRP), Savar Dhaka Bangladesh; ^2^ Department of Physiotherapy and Rehabilitation Jashore University of Science & Technology (JUST) Jashore Bangladesh

**Keywords:** calf muscle, massage gun, myofascial release, trigger points

## Abstract

**Background and Aims:**

Latent trigger points (LTrPs) in the calf muscle contribute to localized pain, increased pain sensitivity, and impaired movement. Massage gun (MG) and myofascial release (MFR) are used to manage LTrPs, but there's limited direct evidence comparing their effects. Therefore, we aimed to compare the effects of MG and therapist‐applied MFR on pain and ankle movement in individuals with calf muscle LTrPs.

**Methods:**

This assessor‐blinded, single‐center randomized clinical trial included 60 participants with calf muscle LTrPs. Participants were randomly assigned to MG (*n* = 30) or MFR (*n* = 30). Both groups received 10 treatment sessions over 5 consecutive days (twice daily) with MG or MFR, followed by a standardized 20‐min ice application. Primary outcomes: Pain intensity was assessed using the visual analog scale (VAS), and pain sensitivity or pressure pain threshold (PPT) was measured with a digital algometer. Secondary outcome: active ankle dorsiflexion ROM (AADF‐ROM) was measured with an inclinometer. Outcomes were measured at baseline and post‐intervention.

**Results:**

Both groups demonstrated significant within‐group improvements in pain intensity, pain sensitivity tolerance, and AADF‐ROM (all *p* = 0.001), with moderate‐to‐large within‐group effect sizes (r = 0.68–0.72). However, between‐group analysis showed significantly greater improvements in the MFR group compared with the MG group across all outcomes, including pain reduction (median change: −2.89 vs −1.69; Z = 6.05, *p* < 0.001, d = 0.92, 95% CI: 0.72–1.14), increased PPT (+1.64 vs +0.62 kg/cm^2^; Z = 5.51, *p* < 0.001, d = 0.85, 95% CI: 0.63–1.03), and greater improvement in AADF‐ROM (+3.01° vs +1.02°; Z = 5.49, *p* < 0.001, d = 0.78, 95% CI: 0.54–0.96), indicating large between‐group effect sizes favoring MFR.

**Conclusion:**

Both interventions effectively improved pain and ankle mobility in individuals with calf muscle LTrPs. However, therapist‐applied MFR yielded superior outcomes and may be the preferred intervention for this musculoskeletal condition.

**Trial Registration:**

Clinical Trials Registry of India (CTRI/2023/08/056794).

**Abbreviations**
AADF‐ROM: Active Ankle Dorsiflexion Range of Motion
LTrPs: Latent Trigger Points
MFR: Myofascial Release
MG: Massage Gun
PPT: Pressure Pain Threshold
VAS: Visual Analogue Scale

## 1 Introduction

Latent trigger points (LTrPs) are a subtype of myofascial trigger points characterized by palpable taut bands within skeletal muscle that are not associated with spontaneous pain but may demonstrate local tenderness or referred discomfort upon palpation [1, 2] Although LTrPs are traditionally described as clinically “silent” at rest, some studies have reported that they may cause localized twitch responses, muscle irritability, and functional impairment, and are increasingly recognized as an important contributor to musculoskeletal dysfunction [3, 4]. Individuals with LTrPs may also experience muscle cramps, reduced joint ROM, altered movement patterns, muscle weakness, and sensory disturbances, all of which may predispose to pain and reduced physical performance. However, the diagnostic validity and clinical manifestations of LTrPs remain partially debated in the literature due to a lack of high‐quality evidence [5, 6].

LTrPs are highly prevalent in the lower extremities, particularly in the calf musculature. Cross‐sectional studies have identified trigger point–like findings in lower‐limb muscles among both symptomatic and asymptomatic individuals, with the gastrocnemius most frequently affected [7]. However, the reported prevalence of LTrPs varies widely across studies, largely due to inconsistent diagnostic criteria, thereby weakening the robustness of epidemiological inferences. University‐based studies also report a higher prevalence of LTrPs in the medial head of the gastrocnemius than in other lower‐leg muscles, regardless of foot posture or activity level [8, 9]. These findings highlight that LTrPs are common even in young, otherwise healthy populations and may represent an underrecognized source of functional limitation. Despite their prevalence, the pathophysiological mechanisms underlying the development of LTrPs remain incompletely understood. Previous evidence suggests that sustained or repetitive muscle overload induces dysfunctional motor endplate activity, leading to persistent sarcomere shortening, impaired local circulation, tissue hypoxia, and increased metabolic demand, thereby contributing to the development of LTrPs [10, 11, 12]. Additional contributing factors include prolonged low‐load activity, eccentric muscle loading, and postural or movement‐related stress, which may perpetuate neuromuscular sensitization and mechanical dysfunction [13, 14]. Although LTrPs do not typically cause spontaneous pain, their presence may compromise muscle efficiency and joint mechanics, thereby contributing to functional decline.

A variety of interventions have been proposed for the management of LTrPs, including manual therapies, needling techniques, and adjunctive physical modalities [10]. Manual approaches such as ischemic compression, transverse friction massage, and myofascial release have demonstrated effectiveness in improving pain and function. Stretch‐based interventions, including proprioceptive neuromuscular facilitation (PNF), may reduce pain and improve ROM in LTrPs. These effects are primarily attributed to neurophysiological mechanisms such as autogenic and reciprocal inhibition, rather than structural changes within the muscle [11, 15, 16]. Additional modalities, such as spray‐and‐stretch, therapeutic ultrasound, and transcutaneous electrical nerve stimulation (TENS), have demonstrated variable efficacy in improving trigger point sensitivity and tissue extensibility [17, 18].

In recent years, device‐assisted soft tissue interventions have gained widespread popularity in clinical and athletic settings. Foam rolling, typically applied as a form of self‐administered static compression, has been shown to produce short‐term improvements in range of motion and physical performance, with proposed mechanisms primarily involving neurophysiological responses, such as altered pain perception and increased stretch tolerance, rather than persistent structural changes in fascial tissue [19]. Similarly, passive soft‐tissue release techniques have demonstrated effectiveness in improving flexibility and reducing perceived tissue stiffness, although current evidence does not consistently support lasting alterations in fascial morphology [20]. Percussive therapy using massage guns is also gaining popularity as a self‐administered or clinician‐assisted modality, with emerging evidence indicating acute benefits on pain modulation, perceived muscle tightness, and range of motion, likely mediated through neuromuscular and sensory mechanisms rather than direct modification of fascial structure [21]. However, despite their growing clinical use, there remains a lack of high‐quality evidence directly comparing percussive massage gun therapy versus therapist‐applied myofascial release, particularly for the management of LTrPs.

This represents clear evidence–practice gap in the conservative management of latent myofascial trigger points. To date, no randomized controlled trial has directly compared the effectiveness of MG and therapist‐applied MFR for reducing pain and improving joint ROM in individuals with lower‐limb LTrPs, particularly in young adults. Therefore, the primary aim of this study was to compare the effectiveness of MG and therapist‐applied MFR in individuals with LTrPs of the calf muscle. The secondary objectives were to describe the sociodemographic characteristics associated with LTrPs among young adults, assess baseline comparability between intervention groups, and evaluate within‐ and between‐group changes in pain and joint ROM from baseline to post‐intervention.

## 2 Methods

### 2.1 Study Design

**Figure 1:**
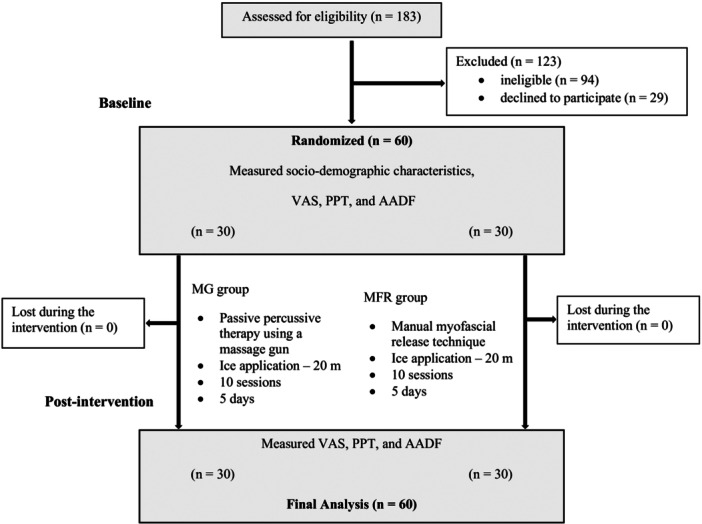
Study flow diagram. AADF, Active Ankle Dorsiflexion; PPT, Pressure Pain Threshold; VAS, Visual Analogue Scale.

This study was conducted as a single‐center, parallel‐group, assessor‐blinded randomized clinical trial designed to compare the effectiveness of MG with MFR for improving LTrPs in the calf musculature. Participants were randomly allocated in a 1:1 ratio to one of two intervention arms. Ethical approval was obtained prior to study commencement, and the trial was prospectively registered on the WHO Trial Registry Platform. The study protocol was subsequently published. Final reporting adhered to the Consolidated Standards of Reporting Trials (CONSORT) guidelines (Figure 1).

### 2.2 Study Settings

The study was conducted at the Outpatient Service Unit of a renowned university in Bangladesh. Participant recruitment, screening, intervention delivery, and outcome assessments took place in this clinical setting between December 2024 and November 2025.

### 2.3 Sample Size

The n4Studies program was used to calculate the sample size for two independent means. Reference values were derived from two randomized controlled trials. The mean standard deviation (SD) of the VAS in the MG group was 2.83 ± 1.24, whereas the trigger point compression or MFR group had a mean VAS ± SD of 3.05 ± 2.27. Assuming an alpha level of 0.05, the system yielded a sample size of at least 25 subjects per group [22]. In the event of a 20% dropout, the final sample size requirement is 30 subjects per group.

### 2.4 Participants

Participants were recruited from individuals attending outpatient physiotherapy services. Eligibility was determined through a structured screening process conducted by a trained physiotherapist.

Inclusion criteria were: (1) age ≥ 18 years [23]; (2) presence of LTrPs in the calf muscle identified by at least two of the following clinical features: hypersensitive tender spot, palpable taut band, local twitch response on palpation, or reproduction of referred pain during compression [9, 24]; and (3) willingness to participate and provide written informed consent.

Exclusion criteria included: (1) diagnosed lower‐limb pathology such as tumor, gout, fracture, rheumatoid arthritis, bursitis, osteoporosis, fibromyalgia, sciatica, or tarsal tunnel syndrome; (2) previous surgery to the affected lower limb or significant anatomical foot deformity; and (3) history of receiving corticosteroid injections more than three times within the previous year [25].

### 2.5 Randomization and Blinding Procedures

Participants were randomly assigned in a 1:1 ratio using a computer‐generated sequence prepared by an independent statistician. Eligible participants were selected through simple random sampling, and allocation concealment was ensured using sequentially numbered, opaque, sealed envelopes (SNOSE) or a secure web‐based system. Study staff received standardized training on enrollment and assignment procedures, and the allocation process was fully documented and monitored for compliance. A single‐blind design was used, in which outcome assessors remained blinded to group allocation, while participants and physiotherapists were aware of the interventions due to practical and resource constraints that precluded blinding. Clear separation of responsibilities was maintained by assigning distinct roles to examiners, therapists, monitoring personnel, and trial supervisors, thereby safeguarding the integrity of the randomization process and minimizing potential performance and detection bias.

### 2.6 Intervention Details

All interventions were delivered by five certified physiotherapists with clinical experience in musculoskeletal rehabilitation. Both groups received 10 treatment sessions over 5 consecutive days (2 sessions per day), each lasting about 30 min. The relatively intensive short‐term treatment schedule was selected to maximize treatment exposure within a controlled experimental period and to facilitate the observation of early neuromuscular, myofascial, and pain‐modulatory responses associated with LTrPs‐targeted interventions. This condensed protocol also enhanced treatment consistency, participant adherence, and supervision while minimizing variability associated with prolonged treatment intervals. To standardize adjunct therapeutic exposure and minimize variability related to post‐intervention soreness, transient inflammatory responses, and pain sensitization, both groups additionally received a standardized 20‐min localized cryotherapy application following the primary intervention [22]. The use of cryotherapy as a common co‐intervention was intentionally incorporated to ensure that any between‐group differences were more likely attributable to the principal interventions rather than unequal analgesic or recovery‐related effects. Participants in both groups were instructed to avoid any additional calf muscle‐targeted therapies during the study period. Exercise adherence and session compliance were monitored through supervised treatment records and participant‐maintained exercise logs.

### 2.7 Massage Gun Intervention (MG Group)

Participants in the MG group received passive percussive therapy with a handheld massage gun (Hyperice, CA, USA) equipped with a 3.8 cm flat massage head. The intervention was applied directly over identified LTrPs in the calf muscle by experienced physiotherapists. Participants were seated comfortably with their legs extended to allow relaxation and optimal access to the target muscle. The massage gun was set to a low speed and applied gently along the muscle fibers. Pain‐sensitive areas indicating LTrPs were treated for 15–30 s per site, with pressure maintained at tolerable levels. Each session consisted of up to 10 min of 53 Hz (1800 rpm/min) percussive therapy based on LTrPs, followed by 20 min of localized cryotherapy application. Treatment parameters were selected based on evidence supporting the neuromuscular and analgesic effects of percussive therapy, while avoiding frequencies insufficient to alter fascia thickness [22, 26].

### 2.8 Myofascial Release Intervention (MFR Group)

Participants in the MFR group received manual myofascial release from experienced physiotherapists with expertise in managing LTrPs. The intervention was applied directly over identified trigger points in the calf muscle. Participants were positioned prone to allow muscle relaxation and optimal access to the target area. Sustained pressure of several kilograms was applied along the restricted myofascial tissue barrier at each trigger point for 90 s per cycle, repeated up to three times until normal muscular tension was restored. Following a maximum 10‐min LTrP‐based release, 20 min of localized cryotherapy were applied. Treatment was administered using the therapist's thumb, with pressure and location adjusted in response to participant feedback, without selecting predetermined trigger point sites [22, 26, 27].

### 2.9 Outcome Measurement

Sociodemographic assessments were conducted only at baseline, before the first treatment session. Primary and secondary outcome assessments were performed at baseline and at the end of the intervention period by blinded assessors using standardized protocols.

### 2.10 Primary Outcomes

Pain intensity was assessed using the Visual Analog Scale (VAS), a validated and reliable subjective measure of pain severity ranging from 0 (no pain) to 10 (worst imaginable pain). It is valid and reliable for pain measurement with an Intraclass Correlation Coefficient (ICC) of 0.97 [28].

Pain sensitivity was assessed using the pressure pain threshold (PPT), measured with a digital pressure algometer applied to the most tender point within the trigger point region. Pressure was increased at a standardized rate until the participant first reported discomfort. Three consecutive measurements were recorded, and the mean value (kg) was used for statistical analysis. The PPT has demonstrated excellent reliability, with test–retest and inter‐rater reliability coefficients of 0.85 [29].

### 2.11 Secondary Outcome

Active ankle dorsiflexion (AADF) ROM was measured using a digital inclinometer with participants in a prone position and the knee flexed to 90 degrees. Participants performed active dorsiflexion three times with standardized rest intervals; the mean value was recorded. Anatomical landmarks and testing procedures were followed in accordance with established guidelines to ensure measurement reliability. The ICC values are 0.86 and 0.83, respectively [30].

### 2.12 Statistical Analysis

Statistical analyzes were performed using IBM SPSS Statistics (version 23.0; IBM Corp., Armonk, NY, USA). Data normality was assessed using the Shapiro–Wilk test, supported by visual inspection of distribution plots (histograms and Q–Q plots). Continuous variables are presented as median and interquartile range (IQR), and categorical variables as frequencies and percentages. Baseline comparability between the two groups was evaluated using the Mann–Whitney U test for continuous variables and Fisher's exact test or the Pearson chi‐square test for categorical variables, as appropriate. Within‐group changes in VAS, PPT, and AADF‐ROM were analyzed using the Wilcoxon signed‐rank test, while between‐group differences in change scores were examined using the Mann–Whitney U test. All analyzes were two‐tailed, and statistical significance was set at *p* < 0.05. Effect sizes were reported as Pearson's r and Cohen's d, with r values of 0.30, 0.50, and 0.60 and Cohen's d values of 0.10, 0.40, and 0.80 interpreted as small, medium, and large effects, respectively, providing standardized measures of intervention effects [31].

### 2.13 Patient and Public Involvement

Patients and/or the public were not involved in the design, conduct, reporting, or dissemination plans of this research.

### 2.14 Ethical Statement

The study was conducted in accordance with the Declaration of Helsinki. Participation was voluntary, written informed consent was obtained from all participants, and they were free to withdraw at any time without affecting their usual care. Ethical approval was obtained from the Ethical Review Committee of the Faculty of Biological Science and Technology, Jashore University of Science and Technology (ID: ERC/FBST/JUST/2023‐171) on 09/05/2023. The trial was also prospectively registered with the WHO Clinical Trials Registry Platform (CTRI/2023/08/056794) on 23/08/2023. Participant confidentiality was maintained, and all data were securely stored.

## 3 Results

### 3.1 Participant Flow and Study Compliance

A total of 60 participants with LTrPs in the calf muscle were enrolled from 183 respondents who met eligibility criteria and were randomly assigned to the MG group (*n* = 30) or the MFR group (*n* = 30). All participants completed the allocated intervention protocol, comprising 10 treatment sessions over five consecutive days. No dropouts were recorded, and outcome data were available for all randomized participants at post‐intervention assessment. Adherence to the intervention schedule was 100% in both groups (Figure 1). No serious adverse events were reported during the intervention period. A small number of participants in both groups reported mild, transient discomfort and post‐intervention soreness, which resolved spontaneously within 24 h and did not interfere with daily activities, indicating good tolerability and safety of both interventions.

### 3.2 Baseline Characteristics

Baseline demographic and clinical characteristics were compatible between the MG and MFR groups (Table 1). The median age was 22 years in both groups, with no statistically significant difference (*p* = 0.941). Gender distribution, education level, living area, body mass index, walking distance, shoe type, and duration of playing did not differ significantly between groups (all *p* > 0.05). These findings confirm adequate baseline homogeneity and support the trial's internal validity, allowing post‐intervention differences to be attributed primarily to treatment effects rather than to baseline imbalance.

**Table 1:** Baseline characteristics.

Variables	MG group (*n* = 30)	MFR group (*n* = 30)	*p*
Demographic characteristics			
Age in years [median (IQR)]^a^	22 (21–23)	22 (20–23)	0.941*
Gender [% (*n*)]^b^			
Male	73.3 (22)	63.3 (19)	0.337*
Female	26.7 (8)	36.7 (11)	
Education [% (*n*)]^c^			
First year	13.4 (4)	23.3 (7)	0.418*
Second year	23.3 (7)	23.3 (7)	
Third year	26.7 (8)	26.7 (8)	
Fourth year	20.0 (6)	20.0 (6)	
Masters	16.7 (5)	6.7 (2)	
Living area [% (*n*)]^b^			
Residential	70.0 (21)	63.3 (19)	0.192*
Non‐residential	30.0 (9)	36.7 (11)	
Clinical characteristics			
BMI [median (IQR)]^a^	24.15 (21.67–25.70)	22.65 (21.15–24.33)	0.360*
Walking distance [% (*n*)]^b^			
More than 1 h	96.7 (29)	93.3 (28)	0.512*
30 min to 1 h	3.3 (1)	6.7 (2)	
Shoe type [% (*n*)]^c^			
Flippers	36.7 (11)	23.3 (7)	0.462*
Normal shoe	46.7 (14)	66.7 (20)	
High‐heeled shoe	16.7 (5)	10.0 (3)	
Duration of playing [% (*n*)]^b^			
More than 1 h	53.3 (16)	40.0 (12)	0.670*
Not playing	46.7 (14)	60.0 (18)	

Abbreviations: BMI, body mass index; IQR, inter quartile range.

^a^ Mann‐Whitney U test.

^b^ Fisher Exact Value and.

^c^ Pearson Chi‐square test.

* baseline compatible.

### 3.3 Effects of Interventions

#### 3.3.1 Pain Intensity (VAS)

Both intervention groups demonstrated statistically significant reductions in pain intensity following the intervention. In the MG group, median VAS scores decreased from 4.00 (IQR: 3.12–4.31) at baseline to 2.31 (IQR: 2.08–3.02) post‐intervention (Z = 5.23, *p* = 0.001, r = 0.68). Similarly, the MFR group showed a greater reduction, with median VAS scores decreasing from 4.82 (IQR: 3.94–5.36) to 1.93 (IQR: 1.42–2.47) (Z = 5.63, *p* = 0.001, r = 0.71). Between‐group analysis revealed a statistically significant difference in favoring the MFR group over the MG group (Z = 6.05, *p* = 0.001), with a large effect size (Cohen's d = 0.92, 95% CI: 0.72–1.14).

#### 3.3.2 Pain Sensitivity (PPT)

Pain sensitivity tolerance improved significantly in both intervention groups. In the MG group, median PPT values increased from 3.74 kg/cm^2^ (IQR: 3.21–4.53) at baseline to 4.36 kg/cm^2^ (IQR: 3.84–5.13) post‐intervention, demonstrating a significant within‐group improvement (Z = 5.37, *p* = 0.001, r = 0.69). The MFR group exhibited a greater increase in PPT values, improving from 3.64 kg/cm^2^ (IQR: 2.83–4.21) to 5.28 kg/cm^2^ (IQR: 4.71–5.82) (Z = 5.67, *p* = 0.001, r = 0.72). Between‐group comparison showed a statistically significant advantage for MFR over the MG group (Z = 5.51, *p* = 0.001), with a large effect size (Cohen's d = 0.85, 95% CI: 0.63–1.03).

#### 3.3.3 Active Ankle Dorsiflexion Range of Motion (AADF‐ROM)

AADF improved significantly in both intervention groups. The MG group showed an increase from a median of 16.94° (IQR: 15.91–18.36) at baseline to 17.96° (IQR: 17.54–19.63) post‐intervention (Z = 5.25, *p* = 0.001, r = 0.68). In contrast, the MFR group exhibited a more pronounced improvement, with dorsiflexion increasing from 16.13° (IQR: 15.27–17.75) to 19.14° (IQR: 18.23–21.08) (Z = 5.54, *p* = 0.001, r = 0.70). Between‐group analysis confirmed a statistically significant difference in favor of the MFR group (Z = 5.49, *p* = 0.001), accompanied by a moderate‐to‐large effect size (Cohen's d = 0.78, 95% CI: 0.54–0.96).

**Figure 2:**
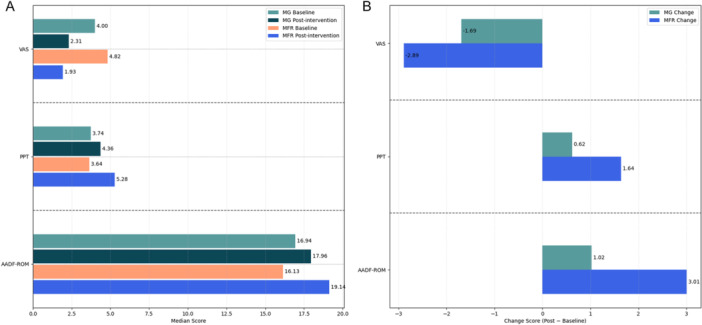
Outcome measures assessments visualization. Figure 2A: Comparison of baseline and post‐intervention median scores for MG and MFR groups. Figure 2B: Comparison of change scores (Baseline to Post‐intervention) between MG and MFR groups.

Overall, while both interventions were effective in reducing pain intensity and improving pain sensitivity and ankle mobility, the MFR group consistently demonstrated superior outcomes across all measured variables compared to MG (Table 2). Figure 2 also shows the visual changes in outcomes over time.

**Table 2:** Outcome measurements.

Outcome group	Baseline median (IQR)	Post‐interventionMedian (IQR)	Within‐group change scores			Between‐group change scores			
			Z	*p*	Effect size (r)	Z	*p*	Cohen's d	95% CI
Pain intensity (VAS)									
MG	4.00 (3.12–4.31)	2.31 (2.08–3.02)	5.23	0.001*	0.68	6.05	0.0001*	0.92	0.72–1.14
MFR	4.82 (3.94–5.36)	1.93 (1.42–2.47)	5.63	0.001*	0.71				
Pain sensitivity tolerance measured by PPT (in kilograms)									
MG	3.74 (3.21–4.53)	4.36 (3.84–5.13)	5.37	0.001*	0.69	5.51	0.0001*	0.85	0.63–1.03
MFR	3.64 (2.83–4.21)	5.28 (4.71–5.82)	5.67	0.001*	0.72				
Active ankle dorsi‐flexion (AADF) ROM									
MG	16.94 (15.91–18.36)	17.96 (17.54–19.63)	5.25	0.001*	0.68	5.49	0.0001*	0.78	0.54–0.96
MFR	16.13 (15.27–17.75)	19.14 (18.23–21.08)	5.54	0.001*	0.70				

Abbreviations: AADF‐ROM, active ankle dorsiflexion range of motion; MFR, myofascial release; MG, massage gun; PPT, pressure pain threshold; VAS, visual analog scale.

* Significant at 95% confidence level; within‐group analysis: Wilcoxon rank test; between‐group analysis: Mann‐Whitney U test.

## 4 Discussion

This randomized clinical trial examined the comparative effects of MG and manual MFR techniques on pain intensity, pain sensitivity (PPT), and ankle dorsiflexion range of motion in individuals with LTrPs of the calf musculature. Both interventions produced statistically significant improvements across all measured outcomes, indicating that soft tissue–focused interventions may positively influence pain and movement‐related impairments associated with LTrPs. However, participants receiving MFR demonstrated comparatively greater improvements across all outcome measures, accompanied by moderate‐to‐large between‐group effect sizes (Cohen's d = 0.78–0.92). These findings suggest that MFR may induce greater short‐term clinical effects than MG under the treatment conditions used in this study, although interpretation should remain cautious given the multifactorial nature of treatment responses and the absence of long‐term outcome assessment.

The reduction in pain intensity observed in both groups is consistent with previous evidence demonstrating that manual and device‐assisted soft tissue interventions can reduce pain and improve symptom perception in individuals with myofascial pain‐related disorders or LTrPs [15, 32]. Importantly, the magnitude of within‐group effects observed in the present study (r = 0.68–0.71) suggests clinically meaningful short‐term changes rather than merely statistically detectable differences. Moreover, the between‐group effect size favoring MFR (d = 0.92, 95% CI: 0.72–1.14) indicates a substantial magnitude of comparative benefit. Nevertheless, the interpretation of these findings requires careful consideration. Contemporary pain science increasingly recognizes that improvements following manual therapy are unlikely to arise from isolated biomechanical corrections or tissue‐specific structural alterations alone [33, 34]. Instead, therapeutic responses are thought to emerge through complex interactions among peripheral sensory input, central pain modulation, affective‐cognitive responses, expectancy effects, contextual factors, and changes in movement‐related confidence and tolerance [34, 35]. Consequently, although MFR produced greater reductions in pain intensity, the present findings should not be interpreted as evidence of a singular mechanistic superiority of manual therapy over percussion‐based interventions. Rather, the observed differences may reflect variations in treatment duration, therapist‐patient interaction, sensory stimulation characteristics, or cumulative neuromodulatory effects associated with sustained manual contact.

The findings related to PPT provide further insight into the potential influence of these interventions on mechanical pain sensitivity. Increased PPT values following intervention indicate reduced sensitivity to externally applied pressure and improved tolerance to mechanical stimulation at the trigger‐point region. While MFR demonstrated comparatively larger improvements than MG (d = 0.85, 95% CI: 0.63–1.03), caution is necessary when interpreting these changes mechanistically. Previous literature has frequently attributed improvements in trigger‐point sensitivity to concepts such as fascial release, local tissue desensitization, or disruption of myofascial adhesions; however, direct evidence supporting these explanations remains limited [10, 36]. Current evidence instead suggests that short‐term changes in PPT are more plausibly associated with altered nociceptive processing, modulation of sensory input, and transient neurophysiological adaptations rather than localized structural modification of soft tissues [33, 36, 37]. Furthermore, studies investigating vibration‐ and percussion‐based therapies have demonstrated inconsistent effects on pressure pain sensitivity, with improvements often dependent on intervention dosage, treatment duration, and participant characteristics [36, 37]. Therefore, the comparatively greater improvements observed following MFR in the present study may reflect a stronger short‐term modulation of pain sensitivity rather than definitive evidence of deeper or more targeted tissue effects.

Significant improvements in active ankle dorsiflexion ROM were also observed in both intervention groups, with greater gains demonstrated in the MFR group (d = 0.78, 95% CI: 0.54–0.96). Reduced ankle dorsiflexion is commonly associated with calf muscle stiffness, altered neuromuscular control, and impaired lower‐extremity biomechanics, potentially contributing to movement inefficiency and altered loading strategies during functional activities [38]. However, interpretation of acute ROM improvements as evidence of structural fascial remodeling, tissue elongation, or reductions in fascial “viscosity” is not strongly supported by current scientific understanding. Emerging evidence indicates that short‐term improvements in flexibility and joint mobility following manual therapy are more likely attributable to increased stretch tolerance, altered perception of discomfort, reduced protective muscle guarding, and transient changes in neuromuscular excitability rather than permanent mechanical deformation of connective tissues [33, 38]. Within this context, the greater ROM gains observed following MFR may reflect enhanced tolerance to end‐range movement and temporary reductions in perceived stiffness rather than lasting morphological changes in fascial structure. This interpretation is more consistent with contemporary models of mobility adaptation and avoids over‐ attribution of structural mechanisms that were not directly measured in the present study.

The present findings also contribute to the evolving understanding of LTrPs within musculoskeletal rehabilitation. LTrPs have been associated with localized tenderness, altered motor activation, increased mechanical sensitivity, and impaired muscle performance [5, 6]. However, the assumption that LTrPs inevitably progress to active trigger points is insufficiently supported in the current literature and should not be considered an established pathophysiological sequence. Accordingly, LTrPs may be more appropriately conceptualized as clinically relevant neuromuscular phenomena associated with altered sensorimotor function and pain sensitization, rather than as definitively progressive pathological lesions. From this perspective, the improvements observed in the present study suggest that targeted soft‐tissue interventions can positively influence symptom‐ and movement‐related impairments associated with LTrPs, even if the precise biological mechanisms remain incompletely understood.

An additional aspect warranting consideration is the intensive intervention dosage used in this study, consisting of 10 treatment sessions administered over five consecutive days. The substantial short‐term improvements observed across outcomes suggest that higher‐frequency intervention schedules may facilitate rapid symptomatic and functional changes in individuals with myofascial pain‐related conditions. This finding aligns with previous rehabilitation literature suggesting that cumulative treatment exposure may influence neuromuscular adaptation, symptom modulation, and movement tolerance [27]. However, the relatively intensive treatment schedule may also partially explain the magnitude of the observed short‐term effects and should therefore be considered when interpreting clinical applicability in routine outpatient practice.

Collectively, the present findings suggest that both MG and MFR may have clinical utility in the short‐term management of calf muscle LTrPs. Nevertheless, MFR consistently demonstrated larger improvements across pain, PPT, and ROM outcomes, indicating comparatively greater short‐term treatment effects under supervised conditions. Importantly, these findings should not be interpreted as establishing categorical superiority of one intervention over another in all clinical contexts. Clinical effectiveness is influenced by multiple factors, including patient preference, therapist expertise, accessibility, treatment adherence, cost, and rehabilitation goals. While MFR may provide greater short‐term therapeutic effects within clinician‐directed rehabilitation settings, MG therapy may still offer meaningful practical value as an accessible adjunctive modality for self‐management, warm‐up, recovery, or maintenance‐based strategies. Accordingly, the present findings support a clinically balanced interpretation in which both interventions may contribute to musculoskeletal rehabilitation, although MFR may offer greater short‐term benefits for individuals presenting with heightened pain sensitivity and mobility restriction.

### 4.1 Strengths and Limitations

This study possesses several methodological strengths. The randomized clinical trial design, complete participant retention, standardized intervention protocols, and blinded outcome assessment strengthen internal validity and reduce the likelihood of systematic bias. In addition, the direct comparison between a therapist‐applied manual intervention and a device‐assisted percussive therapy addresses an emerging and clinically relevant question in musculoskeletal rehabilitation. The inclusion of effect size estimates and confidence intervals further enhances the interpretability and clinical relevance of the findings, beyond reliance on statistical significance alone.

Several limitations should also be acknowledged. First, the absence of long‐term follow‐up limits interpretation regarding the persistence and durability of treatment effects. Consequently, the findings should be interpreted as reflecting only short‐term responses. Second, the study population primarily consisted of young adults, which may reduce generalizability to older individuals, athletes with different training demands, or patients with chronic musculoskeletal disorders. Third, participant and therapist blinding were not feasible given the nature of the interventions, which could introduce performance and expectation bias. Fourth, both groups received standardized cryotherapy, which may have contributed to symptom improvement and limited isolation of the independent effects of MG and MFR. Fifth, although statistically significant between‐group differences and moderate‐to‐large effect sizes were observed, the study did not evaluate minimal clinically important differences (MCIDs), patient satisfaction, or long‐term functional outcomes; therefore, the broader clinical significance of these changes should be interpreted with caution. Finally, psychosocial and behavioral factors such as physical activity level, sleep quality, pain‐related beliefs, and occupational demands were not assessed and may have influenced treatment responses.

Future research should incorporate longer follow‐up periods, multicenter recruitment, broader age ranges, and multidimensional outcome measures to better understand the long‐term effectiveness, clinical relevance, and contextual mechanisms associated with manual and device‐assisted soft tissue interventions.

## 5 Conclusion

Both MG and manual MFR produced significant short‐term improvements in pain intensity, pain sensitivity, and active ankle dorsiflexion ROM in individuals with LTrPs of the calf muscle. Comparatively greater improvements and moderate‐to‐large between‐group effect sizes were observed in the MFR group across all measured outcomes, suggesting that MFR may provide greater short‐term therapeutic benefit within the intervention conditions applied in this study. However, these findings should be interpreted with caution, as the absence of long‐term follow‐up limits conclusions about the persistence and long‐term clinical relevance of the observed effects. From a clinical perspective, the findings support the use of both interventions as potentially beneficial components of conservative musculoskeletal rehabilitation. MFR may be particularly useful in patients presenting with greater pain sensitivity and mobility restriction, whereas MG therapy may offer a practical adjunctive option for self‐management, warm‐up, or supplementary rehabilitation strategies when therapist‐delivered care is less accessible. The findings of this study may assist clinicians in selecting individualized soft tissue interventions based on symptom presentation, treatment accessibility, and rehabilitation goals. Additionally, the study provides a foundation for future investigations into the long‐term effectiveness, optimal treatment dosage, and the combined application of manual and device‐assisted interventions across diverse musculoskeletal populations.

## Author Contributions

**Md Mafrohi Sattar:** conceptualization, methodology, investigation, data curation, writing – original draft, funding acquisition. **Abid Hasan Khan:** methodology, investigation, formal analysis, data curation, software. **Kazi Md Azman Hossain:** methodology, formal analysis, writing – original draft, writing – review and editing, validation. **Md Feroz Kabir:** investigation, data curation, software, resources. **Sharmila Jahan:** investigation, data curation. **K. M. Amran Hossain:** investigation, data curation. **Ehsanur Rahman:** data curation, investigation. **Farzana Sharmin:** methodology, investigation. **Md Saruar Hossain Bhuiyan:** formal analysis, writing – review and editing. **Azharul Islam:** writing – review and editing, visualization. **Md Kabir Hossain:** methodology, supervision, writing – review and editing. **Md Zahid Hossain:** conceptualization, supervision, methodology, project administration, writing – review and editing.

## Ethics Statement

The study was conducted in accordance with the Declaration of Helsinki. Participation was voluntary, written informed consent was obtained, and participants could withdraw at any time without affecting their usual care. Ethical approval was obtained from the Ethical Review Committee of the Faculty of Biological Science and Technology, Jashore University of Science and Technology (ID: ERC/FBST/JUST/2023‐171). Participant confidentiality was maintained, and data were securely stored by the Department of Physiotherapy and Rehabilitation, Jashore University of Science and Technology.

## Conflicts of Interest

The authors declare no conflicts of interest.

## Transparency Statement

The corresponding author (Md Zahid Hossain) affirms that this manuscript is an honest, accurate, and transparent account of the study being reported; that no important aspects of the study have been omitted; and that any discrepancies from the study as planned have been explained.

## Data Availability

The datasets collected and analyzed in this study are available from the corresponding author upon reasonable request. The data are not publicly available because of privacy or ethical restrictions.
